# Circulating miR-200c as a diagnostic and prognostic biomarker for gastric cancer

**DOI:** 10.1186/1479-5876-10-186

**Published:** 2012-09-06

**Authors:** Manuel Valladares-Ayerbes, Margarita Reboredo, Vanessa Medina-Villaamil, Pilar Iglesias-Díaz, Maria José Lorenzo-Patiño, Mar Haz, Isabel Santamarina, Moisés Blanco, Juan Fernández-Tajes, Maria Quindós, Alberto Carral, Angélica Figueroa, Luis Miguel Antón-Aparicio, Lourdes Calvo

**Affiliations:** 1 Medical Oncology Department, La Coruña University Hospital, Servicio Galego de Saúde (SERGAS), As Xubias, 84, La Coruña, PC, 15006, Spain; 2 Translational Cancer Research Lab, Biomedical Research Institute (INIBIC), Carretera del Pasaje, s/n, La Coruña, PC, 15006, Spain; 3 Pathology Department, La Coruña University Hospital, Servicio Galego de Saúde (SERGAS), As Xubias, 84, La Coruña, PC, 15006, Spain; 4 Genomic Group, INIBIC- Biomedical Research Institute (INIBIC), Carretera del Pasaje, s/n, La Coruña, PC, 15006, Spain; 5 Medicine Department, La Coruña University (UDC), Campus de Oza, s/n, La Coruña, PC, 15006, Spain

**Keywords:** Gastric cancer, MicroRNA, miR-200, Blood, Biomarker, Prognostic factors

## Abstract

**Background:**

MicroRNAs are aberrantly expressed and correlate with tumourigenesis and the progression of solid tumours. The miR-200 family determines the epithelial phenotype of cancer cells and regulates invasiveness and migration. Thus, we hypothesised that the quantitative detection of the miR-200 family as epithelial-specific microRNAs in the blood could be a useful clinical biomarker for gastric cancer (GC).

**Methods:**

We initially validated the expression levels of miR-200a, 200b, 200c and 141 in GC cell lines (n = 2) and blood from healthy controls (n = 19) using real-time quantitative reverse transcription PCR (qRT-PCR). The microarray expression profiles of the miR-200 family in 160 paired samples of non-tumour gastric mucosae and GC were downloaded through ArrayExpress and analysed. MiR-200c was selected for clinical validation. The qRT-PCR prospective assessment of miR-200c was performed using 67 blood samples (52 stage I-IV GC patients and 15 controls); the area under the receiver operating characteristic curve (AUC-ROC) was estimated. The Kaplan-Meier and Breslow-Wilcoxon tests were used to assess the correlation of miR-200c with overall and progression-free survival (OS and PFS). Multivariate analyses were performed using the Cox model.

**Results:**

The miR-200c blood expression levels in GC patients were significantly higher than in normal controls (*p =* 0.018). The AUC-ROC was 0.715 (*p* = 0.012). The sensitivity, specificity and accuracy rates of 65.4%, 100% and 73.1%, respectively, were observed. The levels of miR-200c in the blood above the cutoff defined by the ROC curve was found in 17.6% of stage I-II GC patients, 20.6% of stage III patients and 67.7% of stage IV patients (*p <* 0.001). The miR-200c expression levels were not associated with clinical or pathological characteristics or recent surgical procedures. There was a correlation (*p* = 0.016) with the number of lymph node metastases and the increased expression levels of miR-200c in blood were significantly associated with a poor OS (median OS, 9 *vs* 24 months; *p =* 0.016) and PFS (median PFS, 4 *vs* 11 months; *p =* 0.044). Multivariate analyses confirmed that the upregulation of miR-200c in the blood was associated with OS (HR = 2.24; *p =* 0.028) and PFS (HR = 2.27; *p =* 0.028), independent of clinical covariates.

**Conclusions:**

These data suggest that increased miR-200c levels are detected in the blood of gastric cancer patients. MiR-200c has the potential to be a predictor of progression and survival.

## Background

Gastric cancer (GC) is among the most frequent types of cancer worldwide [1] with a total of 989,600 new cases and 738,000 deaths estimated in 2008. Although GC rates have decreased in recent decades, there are significant regional variations in incidence and the rates for the gastro-oesophageal junction and cardiac adenocarcinomas have increased in several Western countries. In Spain, the adjusted mortality rates were 13 per 100,000 males and 5.5 per 100,000 females. In Galicia, in the northwest of Spain, the mortality rates are even higher, reaching 16.14 per 100,000 in males. Global survival rates are poor, lower than 28% at 5 years [2].

The stage at diagnosis and the options for curative surgery remain the most important prognostic factors. However, distant and loco-regional relapses frequently occur in spite of resection and multimodality therapy. Well-characterised biomarkers are necessary for early diagnosis, to predict metastatic progression and to personalise therapy. Nevertheless, the currently available blood tumour markers are not recommended for the screening or diagnosis of GC, do not have independent prognostic value and are not recommended for prognosis or prediction [3].

Haematogenous tumour seeding is considered an early event in the metastatic process. Therefore, the detection of circulating tumour cells (CTC) could be useful to identify the patients at a high risk of disease progression and death and might indicate the need for further therapeutic approaches [4]. The PCR amplification of tissue or tumour-selective cellular and circulating nucleic acids (CNA) is the most powerful tool for the detection of CTC or occult metastases [4,5].

Mature microRNAs are single-stranded, noncoding RNAs that play key roles in various cellular processes commonly implicated in cancer, such as differentiation, cell growth, angiogenesis, epithelial-to-mesenchymal transition (EMT) and invasion. A large amount of data has revealed the correlation between specific tumours and differential miRNA expression profiles, thus providing a new class of disease-specific biomarkers [6-8]. An increasing number of studies analysing the miRNA expression profiles in gastrointestinal tumours, including GC and their potential clinical relevance have been reported [9,10]. The content for a given miRNA species is estimated at 10^3^ to 10^4^ molecules per cell, which is one to two orders of magnitude more than most mRNAs [11]. Both messenger and non-coding RNAs can be detected in blood and studies indicate that miRNAs are particularly stable and abundant [12-15]. Circulating miRNAs could be derived from passive leakage from apoptosis or necrosis of cancer cells but also from tissue damage or chronic inflammation. In addition, both cancer and nonmalignant cells, including immune cells, can actively release miRNAs, either microvesicles-associated or free, in a selective manner [16].

Developmental [17,18] and expression profiles studies [19,20] show an enrichment of the miR-200 family in differentiated epithelial tissues. It has been suggested that the miR-200 family is a powerful marker and an essential regulatory factor of the cancer cell epithelial phenotype [21-25]. The miR-200 family of miRNAs consists of five members: miR-200a, 200b and 429, located on chromosome 1p36; and miR-200c and 141, located on 12p13. MiR-200a and miR-141 share a seed sequence, while miR-200b, miR-200c and miR-429 also share a seed sequence, which differs from that of miR-200a/141 by one nucleotide. However, there is evidence that the different miRNAs could control different regulatory networks [26,27]. Previous reports have indicated that the levels of peripheral blood-derived exosomal miR-200c are increased in ovarian cancer patients [28] and the serum levels of miR-141 are specifically elevated in prostate cancer patients [13,29]. Both miR-200a and miR-200b are significantly elevated in the sera of pancreatic cancer and chronic pancreatitis patients compared with healthy controls [30].

Therefore, we hypothesised that the quantitative detection of the miR-200 family, as epithelial-specific miRNAs, in the whole blood could be useful as clinical biomarkers in gastric cancer patients. Therefore, the blood miR-200 cluster expression might correlate with GC diagnosis, staging and prognosis. Our results demonstrated that miR-200c expression levels were increased in the blood of GC patients. Likewise, the blood levels of miR-200c emerged as a compelling and independent prognostic indicator for the progression and survival of GC patients.

## Methods

### Participants

Consecutive GC patients from the Medical Oncology Unit at the University Hospital in La Coruña (Galicia, Spain) were eligible for the study. The inclusion criteria included a confirmed pathological diagnosis of gastric or gastro-oesophageal junction adenocarcinoma and no prior systemic medical therapy for cancer. The exclusion criteria included any other previous malignancy, coagulation disorders and a platelet count less than 20.0 x 10^9^ L^-1^.

The diagnostic work-up included a clinical examination, blood sampling, endoscopy (when clinically indicated) and computed tomography (CT) scanning of the chest, abdomen and pelvis. The patients were followed up clinically with imaging every 8 to 12 weeks for the first 2 years and every 6 months thereafter to monitor disease progression.

In GC patients, peripheral venous blood (PB) for quantitative reverse transcription PCR (qRT-PCR) analysis was obtained after surgery or in the presence of clinical and radiological disease when surgery was not indicated. The first 5 mL of collected blood was discarded to avoid contamination with epidermal cells. The PB (10 mL) was collected in EDTA-containing tubes. Then, the PB was frozen at −20°C in RNA*later* for storage until RNA extraction.

The controls were recruited from the patients’ family and relatives. We only excluded subjects with a previous history of malignant disease. Thus, controls with different chronic but stable diseases (e.g., peptic disease, hypertension, diabetes mellitus or heart disease) were eligible and consecutively recruited. The control cohort was selected to include a sex and age distribution that was comparable to the patient group.

This study was approved by the Ethics Committee of Clinical Investigation of Galicia (Spain) and conducted in compliance with the Helsinki Declaration. Written informed consents were obtained from all the patients and the controls prior to their inclusion in the study.

### Pathological analyses

Tumours and regional lymph nodes collected during surgery were processed on a routine diagnostic basis. Histological type, depth of invasion and nodal involvement were analysed and the disease was staged and graded according to the TNM and Laurent classification [31]. Residual disease status at the time of blood sampling was classified as R0 when no residual disease was present after surgery, R1 when microscopic residual disease was found and R2 in the presence of macroscopic disease. The patients from whom the blood was obtained before the start of neo-adjuvant treatment were categorised as R2. When surgery was not performed, the pathological diagnosis was based on endoscopic or radiological-guided biopsies.

### Blood microRNA isolation and qRT-PCR

To isolate the miRNA fraction, the RiboPure-Blood Kit was used with the alternate protocol: isolation of small RNAs (Applied Biosystems, Foster City, CA, USA). The procedure was performed using 0.5 ml of whole blood per preparation. The absorbances at 260/280 and 260/230 were assessed using a NanoDrop™ 1000 spectrophotometer (Nanodrop Technologies, Wilmington, DE, USA). The purified RNA was further processed using qRT-PCR or stored at −80°C until use.

Reverse-transcription (RT) PCR was performed with 25 ng (up to 6.6 μl) of total RNA using the mirVana™ qRT-PCR miRNA Detection Kit (Ambion, AM1558) with 2 μl 5X RT Buffer, 1 μl 1X RT Primer (Ambion, miR-200a, A30094; miR-200b, AM30095*; miR-200c, AM30096*; miR-141, AM2052*) and 0.4 μl of ArrayScript Enzyme Mix for a total volume of 10 μl.

For the PCR reaction, 10 μl of RT reaction and PCR Master Mix were used. The PCR Master Mix consisted of 5 μl 5X PCR buffer containing SYBR Green I, 0.2 μl SuperTaq 5 U/μl, 0.5 μl PCR primers and 9.3 μl of nuclease-free water for a total volume of 15 μl. Real-time PCR was performed on the LightCycler® 480 Instrument (Roche, Mannheim, Germany).

To control input variability and sample normalisation, primer sets specific for the small RNA species U6 snRNA (Ambion, AM30303) and 5S rRNA (Ambion, AM30302) were used. These primer sets were used not only as internal controls but also to verify the integrity of the RNA and the reverse transcription reaction. Any specimen with inadequate U6 snRNA or 5S rRNA expression would be excluded from the study.

For miR-141, miR-200b and miR-200c, the PCR cycling conditions and analysis were as follows: denaturation at 95°C for 8 seconds; cycling, 40 cycles of 95°C for 5 seconds, 60°C for 5 seconds and 72°C for 2 seconds; melting curve analysis, 1 cycle at 95°C for 5 seconds, 55°C for 1 minute 5 seconds and 95°C continuous; and finally, cooling at 40°C for 10 seconds. The conditions were identical for miR-200a, U6 snRNA and 5S rRNA, except the denaturation step was 1 cycle at 95°C for 6 seconds.

We verified that the amplification of each PCR product was specific using a melting curve analysis. The amplification efficiency was determined for both target and reference genes. Each assay was performed at least in triplicate. The quantification cycle (Cq) was performed using LightCycler 480 Quantification software (Roche, Mannheim, Germany). For further data analysis, only those miRNAs with a Cq value equal to or below 35, representing detection of one single-molecule template [32], were considered. Positive and negative controls were included in each experiment.

The Relative Expression Software Tool (REST) was used to analyse the relative miRNA expression in each sample and to determine the fold difference for every miRNA [33]. The expression levels of the target miRNAs were standardised using an index containing 5S rRNA and U6 snRNA.

miRNA analyses were performed with no knowledge of the clinical or follow-up data.

### miR-200 cluster expression profiling

To analyse the expression of the miR-200 family in gastric cancer, the OE19 and MKN-45 human gastric cell lines were used. The cell lines were maintained in Dulbecco’s modified Eagle’s medium (DMEM) with high glucose and MegaCell^TM^ RPMI-1640 medium (both provided by Sigma–Aldrich Química, Madrid, Spain) supplemented with 10% inactivated foetal calf serum, 1% penicillin, 1% streptomycin and 1% amphotericin at 37°C in 5% CO_2_. The cells were recovered with 1% trypsin–1% EDTA cell-dissociating reagent.

The isolation of total RNA (including miRNA) from the cell cultures was performed using the mirVana^TM^ miRNA isolation kit (Ambion, Inc. AM1560). The procedure was performed using 10^7^ cultured cells at 70% confluence.

The miR-200 expression profiles in paired samples (n = 160) of non-tumour gastric mucosa and GC were obtained using bioinformatic analysis of the data originally published by Ueda T, et al. [10]. The microarray expression was downloaded through the public repository ArrayExpress (experiment number E-TABM-341. http://www.ebi.ac.uk/arrayexpress/). Only the normalised expression values were used for subsequent analysis. The differential expression levels were calculated using a moderate *t*-test implemented in the Bioconductor *limma* package (R statistical software). The comparisons were performed using *t*-test and pairwise *t*-tests. The resulting *p* values were adjusted for multiple testing using Benjamini-Hochberg’s adjustment [34,35].

### Study design and statistical analyses

The primary aims were to estimate the diagnostic accuracy and usefulness of miRNA as measured by qRT-PCR in the blood of GC patients as a clinical biomarker and to determine its potential prognostic value. The study was performed following the proposed guidelines of the Early Detection Research Network [36]. The design and results are presented in accordance with the REMARK [37] and MIQE guidelines [38].

The receiver operating characteristic (ROC) curve was constructed by plotting sensitivity (Y-axis) *vs* 1-specificity (X-axis) and the areas under the curve (AUC) were calculated. The diagnostic performance including sensitivity, specificity, positive and negative predictive values and accuracy of miR-200c quantification was also estimated [36]. The potential correlation among blood miRNA levels and the clinical and pathological features of the study subjects were analysed. The normality of the distribution of miRNA expression was analysed using the Kolmogorov-Smirnov test. Thus, parametric or non-parametric statistics were used, as appropriate. The relationships between miR-200c levels and the quantitative clinical variables were analysed using the Pearson correlation.

Progression-free survival (PFS) was measured as the time between the baseline blood sampling for miRNA analysis and the documentation of first tumour progression, based on clinical and radiological findings, or death (events). Overall survival (OS) was measured from the time at which the baseline blood sample was obtained to the date of death from any cause or date of last follow-up. The patients who were alive and progression-free at the time of analysis were censored by using the time between the blood assessment and their most recent follow-up evaluations. The distributions of time-to-event end points, namely PFS and OS, were estimated using the Kaplan-Meier method and compared using the Breslow-Wilcoxon test.

Multivariate survival analyses (PFS and OS) were performed using Cox regression models. We estimated hazard ratios (HRs), 95% CI and *p* values. All statistical tests were two-sided and *p* values less than 0.05 were considered significant. SPSS Statistics 19.0 for Windows (IBM Corporation, Armonk, NY, USA, 2011) and Graph Pad Prism 5 (GraphPad Software, La Jolla, CA, USA, 2007) were used for data analyses.

## Results

### The miR-200 family of microRNAs was highly expressed in gastric cancer

**Figure 1:**
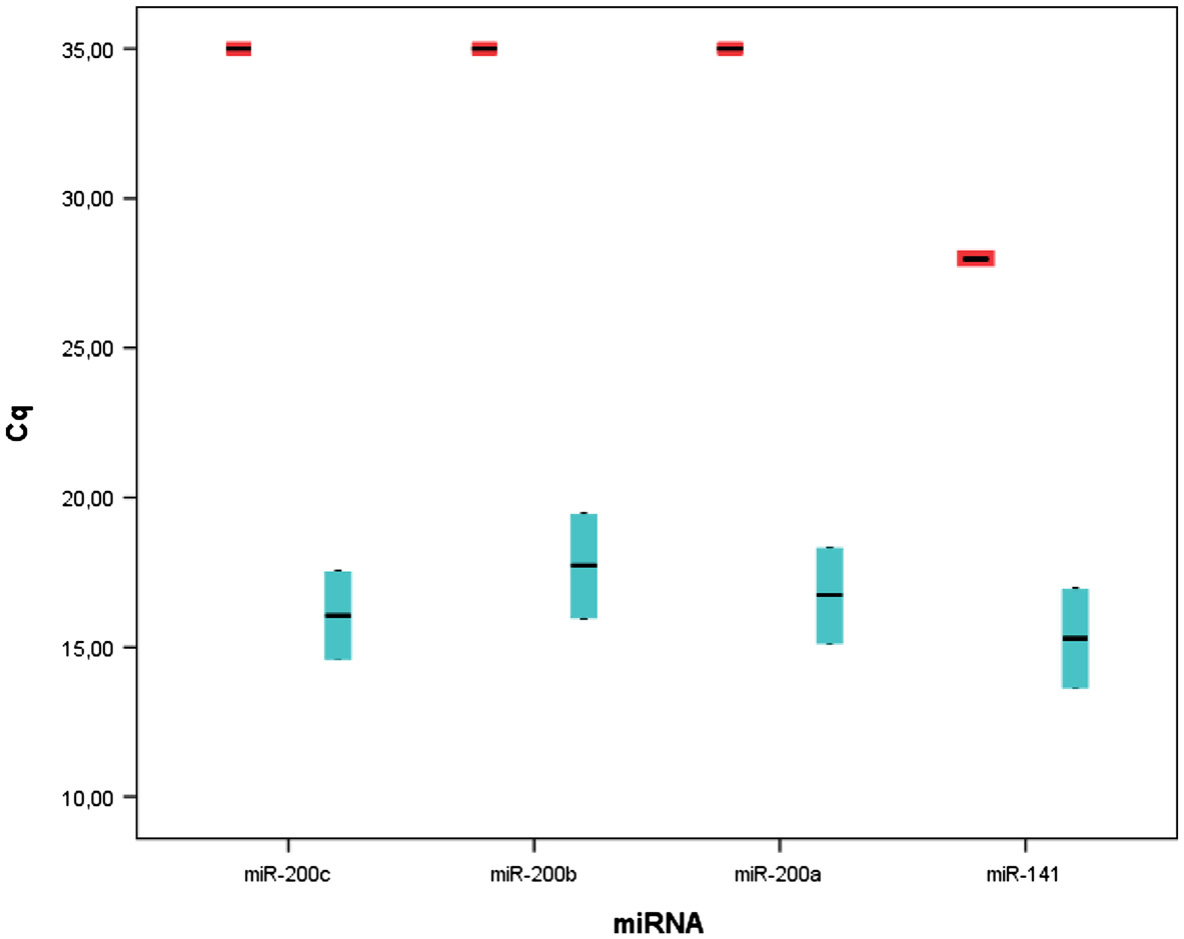
**Real-time PCR of the miR-200 family in control blood and gastric cancer cell lines.** The raw quantification cycle (Cq) data for the miR-200 cluster in the control blood samples (n = 19) and gastric cancer cell lines (OE-19 and MKN-45) are depicted. In the blood, the mean Cq was lower for miR-141 (Cq = 28) compared with those for miR-200a (Cq = 35), 200b (Cq = 35) and 200c (Cq = 35; ANOVA, *p* < 0.001; Bonferroni post hoc test, *p* < 0.001). In the GC cell lines, the mean Cqs were 15.3, 16.7, 17.7 and 16.1 for miR-141, 200a, 200b and 200c, respectively, without significant differences (ANOVA, *p* = 0.133). The red boxes indicate control blood samples, while the light-blue boxes indicate the gastric cancer cell lines.

To investigate the differential expression levels of the miR-200 cluster, we used real-time PCR to analyse the expression levels of miR-200a, 200b, 200c and miR-141 in total RNA extracted from the GC cell lines OE-19 and MKN-45. We compared the miRNA expression profiles, calculated using REST as described, with those of normal human blood (a control group consisting of pooled RNA obtained from 19 healthy donor blood samples). The relative expression ratios of every target miRNA were significantly higher in the GC cell lines compared with the control blood. In OE-19 cells, the miRNAs were upregulated by a mean factor of 6.61x10^5^, 9.99 x10^3^, 4.47 x10^5^ and 2.54 x10^5^ for miR-200c (*p <* 0.001), 141 (*p =* 0.018), 200a (*p <* 0.001) and 200b (*p <* 0.001), respectively. In MKN-45 cells, the miRNAs were upregulated by a mean factor of 4.94 x10^5^, 5.79 x10^3^, 2.86 x10^5^ and 1.30 x10^5^ for miR-200c (*p = 0*.033), 141 (*p <* 0.001), 200a (*p <* 0.001) and 200b (*p <* 0.001), respectively. Thus, the highest fold-change observed in the GC cell lines relative to control blood was 5.78 x10^5^ for miR-200c. In addition to the miRNA expression data analysis obtained by REST, we compared the raw Cq data for every miRNA in the control blood and gastric cancer cell lines. In the blood, the mean Cq was lower for miR-141 (Cq = 28) compared with miR-200a (Cq = 35), 200b (Cq = 35) and 200c (Cq = 35). These differences were significant (ANOVA, *p <* 0.001; Bonferroni *post hoc* test, *p <* 0.001) suggesting an increased background miR-141 expression in non-tumour blood relative to the other miR-200 family members. In the GC cell lines, the mean Cqs were 15.3, 16.7, 17.7 and 16.1 for miR-141, 200a, 200b and 200c, respectively, without significant differences (ANOVA, *p =* 0.133; Figure 1).

To ascertain whether the miR-200 cluster signature differs between GC and non-tumour mucosa and between the two histological subtypes of GC, the expression profiles were retrieved from Ueda et al. [10]. We observed that the miR-200 family was not differentially expressed in the paired non-tumour mucosa and cancer samples. Furthermore, miR-141, 200a, 200b and 200c were not differentially expressed between the GC histological subtypes (diffuse and intestinal). Additional file 1: Figures S1, S2 and Additional File 1: Tables S1, S2.

### Patients and clinical data

From November 2006 to July 2010, 52 patients with histologically proven GC were consecutively recruited for this study. The clinical characteristics of the patients are shown in Table 1. The control cohort included 15 cases. The mean age was 65.3 years (standard error of the mean [SEM], 1.9; range, 49 to 74 years) in the control group and 65.9 years (SEM, 1.32; range, 43 to 85) in the patient group (*t* test, *p =* 0.82). The ratio of males to females was similar among the controls and the patients (Yates-corrected χ^2^, *p =* 0.07).

**Table 1:** Patient characteristics (n = 52)

**Characteristic**	**n**	**%**
**Median (range) age, yrs**	65.9 (42–85)	
**Gender**		
** Women**	10	19
** Men**	42	81
**ECOG**		
** 0-1**	37	71.2
** 2**	10	19.2
**Location**		
** Proximal, upper third**	13	25
** Distal**	36	69.2
** Multicentric**	3	5.8
**Stage**		
** I-II**	9	17.3
** III**	12	23.1
** IV**	31	59.6
**Lymph Nodes**		
** Negative**	9	17.3
** Positive**	24	46.2
**Histological type**		
** Intestinal**	28	53.8
** Diffuse**	21	40.4
** Mixed**	3	5.8
**R Status**		
** R0**	20	38.5
** R1-R2**	32	61.5
**Grade**		
** Low**	21	40.4
** High**	27	51.9
**Vascular / Perineural Invasion**		
** Unknown**	22	42.3
** No**	11	21.2
** Yes**	19	36.5

Abbreviations: ECOG: Eastern Cooperative Oncology Group performance status. Residual Status (R): R0, no residual tumour; R1-2 microscopic or macroscopic residual tumour.

The blood was obtained after R0 surgery in 20 patients (38.5%). In 32 patients, the blood samples were obtained in the presence of residual or metastatic disease, both of which were categorised as R2 at the time of blood sampling. In the patients receiving surgery (71.2%; 37/52), the number of lymph nodes analysed was 19 (range, 0–76; St. D, 16.2). Chemotherapy was administered to 44 patients (84.6%).

All patients were followed until death or study completion. The last date of follow-up for the survivors was September 5, 2011. Disease progression events occurred in 38 patients (73.1%). The median PFS was 6 months (95% CI, 1.4 to 10.6 months). There were 7 relapses among stage I–III patients and 31 progressions of metastatic disease. The median OS was 15 months (95% CI, 11.1 to 18.9 months) and 35 patients (67.3%) died of advanced disease. Most of the PFS events (29/38; 76.3%) and OS (18/35; 51.4%) events occurred in the first 9 months of follow-up. The mean (SEM) follow-up time for the patients still alive at the time of the analysis was 26.3 (3.7) months (median, 24 months; range, 6 to 53 months).

### Expression of miRNA in blood samples

As described above, we found that miR-200c was not only upregulated in GC cell-lines compared with control blood, it was expressed at the highest levels of all miR-200 family members. Thus, miR-200c was selected for clinical validation.

**Figure 2:**
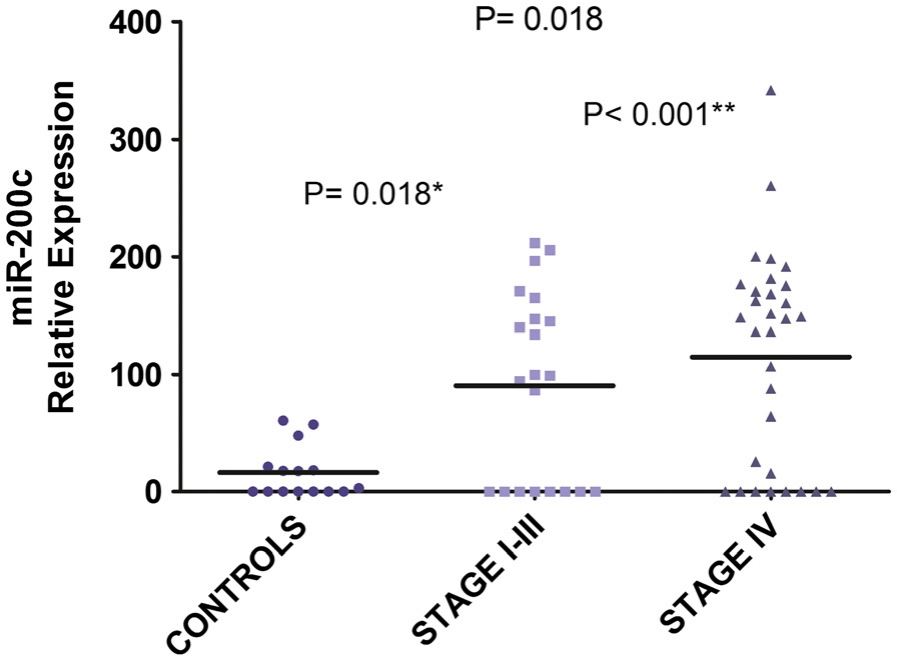
**Real time PCR of miR-200c in blood samples.** The graph depicts the increasing relative expression levels for the mean blood expression levels of miR-200c (Kruskal-Wallis test, *p* =0.018) from controls (n = 15) and gastric cancer samples (n = 52). Significant differences were observed between the blood expression levels of miR-200c in each TNM stage subgroup and the control group (Bonferroni *post hoc* test: stage I-III *vs* control, *p = 0.018; stage IV *vs* control, **p < 0.001). MiR-200c was measured in triplicate using qRT-PCR and normalised to U6 snRNA and 5S rRNA. The horizontal bar denotes the mean value for each group.

Real-time quantitative assessment of miR-200c was performed using 67 blood samples (52 patients and 15 controls). The mean relative miR-200c expression (Figure 2) was 16.2 (SEM, 5.6; CI 95%, 4.1 to 28.3) in controls, 90.3 (SEM, 17.4; CI 95%, 53.9 to 126.6) in stage I-III patients and 114.6 (SEM, 16.3; CI 95%, 81.4 to 114.9) in stage IV GC patients (*p =* 0.018; Kruskal-Wallis test. Bonferroni *post hoc* test: stage I-III *vs* control, *p =* 0.018; stage IV *vs* control, *p* < 0.001). The confidence interval with the alpha level of significance at 99% estimated using the Monte Carlo test was 0.015 to 0.022.

**Figure 3:**
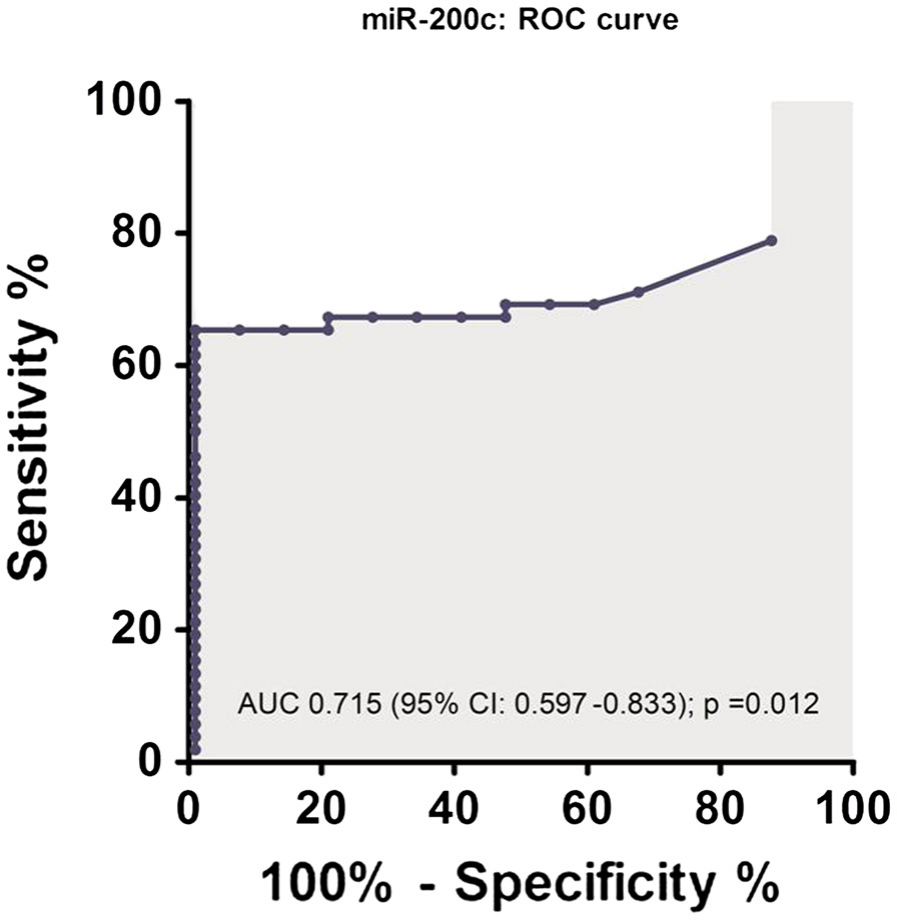
**The role of blood miR-200c in gastric cancer diagnosis.** The receiver-operating characteristic (ROC) curve analysis using blood miR-200c expression levels for discriminating gastric cancer (n = 52) and controls (n = 15) is shown. The area under the ROC curve is shown [AUC 0.715 (95% CI, 0.597–0.833); *p* = 0.012; cutoff value is 62.4; sensitivity, 65.4%; specificity, 100%].

An ROC curve was constructed (Figure 3). Comparing the relative miR-200c levels in controls and patients, the AUC was 0.715 (95% CI, 0.597–0.833; *p* = 0.012). According to the ROC curve, the relative blood level of miR-200c of 62.4 was defined to be the optimal cutoff value for differentiating GC patients and controls (Youden's index). With this cutoff value for miR-200c, the sensitivity, specificity, positive and negative predictive values and accuracy values of 65.4% (95% CI, 50.8 to 77.7), 100% (95% CI, 74.7 to 99.4), 100% (95% CI, 87.4 to 99.7), 45.5% (95% CI, 28.5 to 63.4) and 73.1% (95% CI, 60.7 to 82.9), respectively, were achieved. The relative expression values for miR-200c in blood above this cutoff point were found in 17.6% of stage I-II patients, in 20.6% of stage III patients and in 67.7% of stage IV GC patients (*p <* 0.001; exact test). These findings suggested that elevated blood miR-200c could be detected in the early stages of GC and therefore facilitate early disease detection.

### Clinical and pathological characteristics and miR-200c levels in blood

The clinical and pathological characteristics and the miR-200c expression levels in the blood from cancer patients are shown in Table 2. The relative expression levels for miR-200c in the blood of GC patients were not associated with any of the parameters analysed. Furthermore, the percentage of patients with miR-200c levels above its mean value (mean, 104.8; SEM, 12) was not associated with clinical and pathological characteristics.

**Table 2:** The distribution of clinical and pathological parameters and the levels of miR-200c in blood

**Parameter**	**n**	**miR-200c mean (SEM)**	***p *value**	**High miR-200c n (%)**	***p *value**
**Age (y)**			0.985		0.0895*
** < 70**	32	100.3 (14.1)		17 (53.1%)	
** ≥70**	20	112 (21.9)		11 (55%)	
**Gender**			0.692**		1***
** Male**	42	107.3 (13.5)		23 (54.8%)	
** Female**	10	94.2 (26.8)		5 (50%)	
**Location**			0.742**		1***
** Proximal, upper third**	13	120.0 (31.1)		7 (53.8%)	
** Distal**	36	98.5 (12.8)		19 (52.8%)	
** Multicentric**	3	114.2 (58.3)		2 (66.7%)	
**Stage**			0.211		0.191*
** I-III**	21	90.3 (17.4)		9 (42.9%)	
** IV**	31	114.6 (16.3)		19 (61.3%)	
**pT**			0.683		0.693***
** pT1-T2**	18	93.2 (31.6)		13 (72.2%)	
** pT3-T4**	16	95.9 (17.6)		13 (81.3%)	
**pN**			0.516		0.259***
** Node Negative**	9	67.4 (28.4)		3 (33.3%)	
** Node Positive**	24	115.9 (18.6)		14 (58.3%)	
**Histological type**			0.179		0.246*
** Intestinal**	28	86.5 (15.1)		13 (46.4%)	
** Diffuse**	24	126.2 (18.5)		15 (62.5%)	
**ECOG**			0.263		0.481***
** 0-1**	37	94.2 (15.5)		20 (54.1%)	
** 2**	10	147.1 (56.2)		7 (70%)	
**Residual disease (R)**			0.113		0.312*
** R0**	20	89.7 (18.2)		9 (4%)	
** R1-2**	32	120.4 (27.9)		19 (59.4%)	
**Number of Metastatic sites**			0.551**		0.753***
** 0**	23	95.4 (16.5)		11 (47.8%)	
** 1**	21	113.2 (21.1)		12 (57.1%)	
** ≥2**	8	109.8 (31.1)		5 (62.5%)	
**Grade**			0.405		0.146*
** Low**	21	73.6 (20.9)		8 (38.1%)	
** High**	27	116.9 (23.1)		16 (59.3%)	
**Vascular / Perineural Invasion**			0.914		0.705*
** No**	11	103.3 (22.8)		5 (45.5%)	
** Yes**	19	97.5 (22.8)		10 (52.6%)	
**Neutrophils (10**^**-9**^**/L)**			0.705		0.696*
** ≤ 7.5**	39	104.4 (13.1)		22 (56.4%)	
** >7.5**	12	114.9 (29.4)		6 (50%)	

The miR-200c relative expression levels (REL) are shown in arbitrary units. The levels of miR-200c were considered high when the REL was above the mean. Residual disease (R) was categorised as R0 when no residual disease was present and as R1-2 when microscopic or macroscopic residual disease was found. Mann–Whitney test. * Pearson χ2. **Kruskal-Wallis test. *** Fisher's exact test.

To explore the possible influence of recent surgical procedures on the circulation of miRNA, we analysed miR-200c levels according to the time interval from surgery and blood sampling. The median time from surgery to blood sampling for miRNA quantification was 6 weeks (mean, 19.1 weeks; SEM, 5.5; range, 2 to 155 weeks). There were no significant differences in miR-200c levels according to time intervals (< 6 or ≥ 6 weeks) from the last surgery adjusted for tumour stage (ANOVA, *p =* 0.284).

### Prognostic significance of miR-200c levels in blood

The correlations of potential prognostic factors and miR-200c levels in the blood in gastric cancer patients are shown in Table 3. There was only a significant correlation (Pearson’s r = 0.438, *p* = 0.016) between miR-200c levels and the number of lymph node metastases.

**Table 3:** The correlations of prognostic factors and miR-200c levels in the blood of gastric cancer patients

	**n**	**r**	***p*value**
**Weight loss (%)**	51	0.082	0.568
**Number of positive lymph nodes**	30	0.438	0.016
**LDH**	52	- 0.023	0.872
**Albumin**	51	- 0.130	0.365
**Alkaline Phosphatase**	52	- 0.041	0.770
**Neutrophil counts**	51	0.132	0.356

Computed using the Pearson correlation test.

To generate survival curves, we converted continuous miR-200c expression levels measured using qRT-PCR to a dichotomous variable, using its mean levels of expression as a threshold (10). Using this approach, miR-200c was overexpressed in the blood of 53.8% (28/52) of patients. The mean values (with SEM) in the low and high expression groups for miR-200c were 23.9 (7.9) and 174.1 (8.5), respectively (Mann–Whitney test, *p <* 0.001). The percentage of patients with miR200c overexpression tended to increase with TNM stage: 33.3% (3/9) in stage I-II patients, 50% in stage III patients (6/12) and 61.3% (19/31) in stage IV patients (*p =* 0.076).

**Figure 4:**
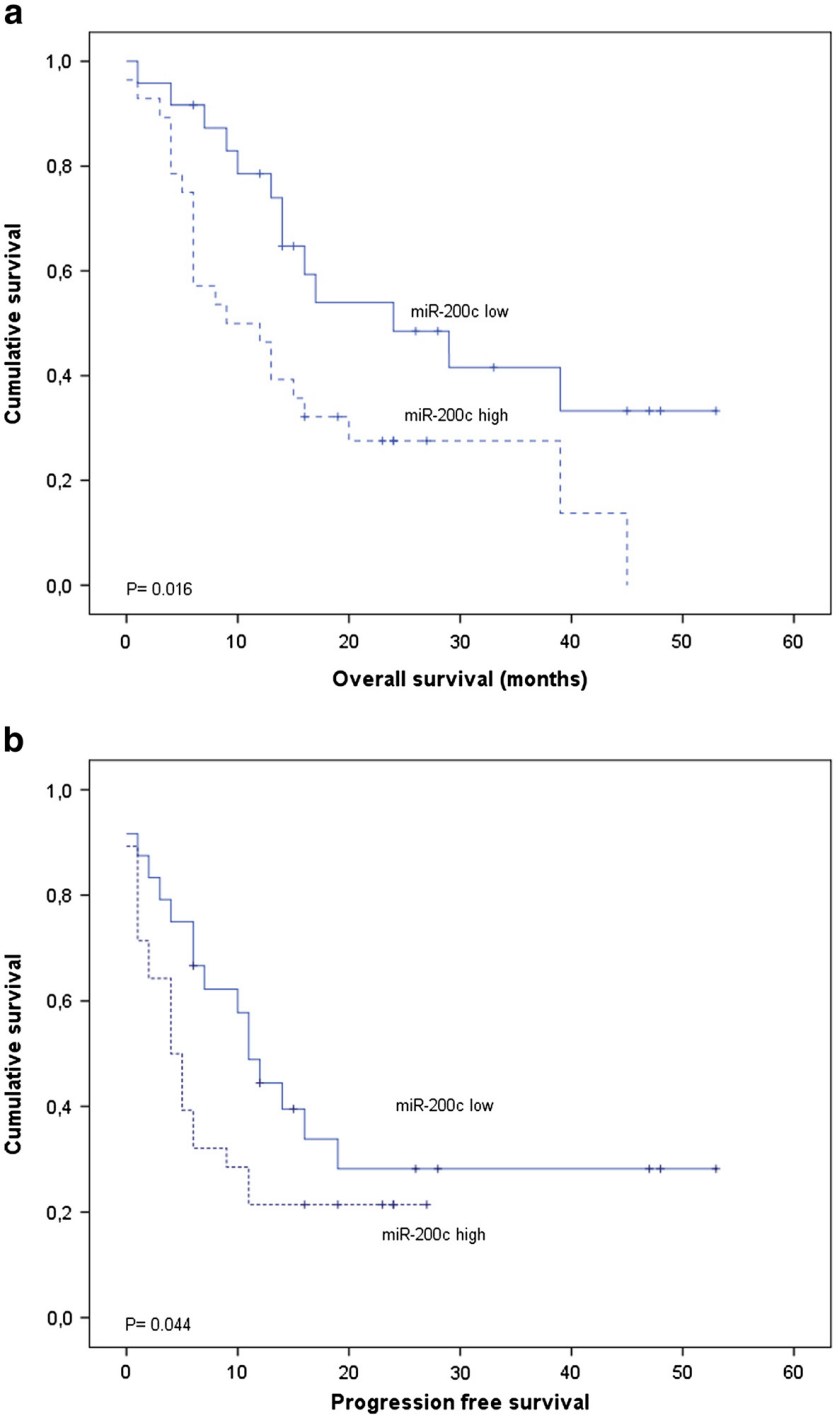
**miR-200c expression levels measured in the peripheral blood are associated with poor prognosis in gastric cancer patients.** Kaplan-Meier curves showing (**a**) the overall survival (OS) and (**b**) the progression-free survival (PFS) of 52 subjects with high or low blood expression levels of miR-200c. Continuous miR-200c expression levels measured using qRT-PCR were converted to a dichotomous variable using the mean level of expression as a threshold. The *p* values were computed using the Breslow-Wilcoxon test.

The Kaplan-Meier curves for patient OS and PFS categorised according to miR-200c expression levels in the blood are shown in Figure 4. The increased blood expression of miR-200c was significantly associated with a poor overall survival (Breslow-Wilcoxon text; *p =* 0.016). The median and mean OS for the group with high miR-200c expression levels were 9 months (95% CI, 1.7–16.3) and 17.4 months (95% CI, 11.2–23.6), respectively. In the group with low miR-200c blood expression levels, the median OS was 24 months (95% CI, 8.1–39.9) and the mean OS was 29.2 months (95% CI, 20.9–37.6).

With regards to PFS, the median estimate for those patients with low levels of miR-200c in the blood was 11 months (95% CI, 7.9 to 14.1). In contrast, the median PFS was 4 months (95% CI, 1.8 to 6.2) in patients with high miR-200c levels (Breslow-Wilcoxon test; *p =* 0.044).

The relative strength of blood expression levels of miR-200c as an independent prognostic factor was evaluated by performing a Cox multivariate analysis. The details of this analysis are listed in Table 4. With the inclusion of miR-200c expression levels in the model, the independent predictors of PFS were as follows: stage IV disease (HR = 5.52; *p =* 0.005), residual disease (HR = 4.29; *p =* 0.023); and miR-200c overexpression (HR = 2.27; *p =* 0.028). Similar results were achieved for the prediction of OS including stage IV disease (HR = 8.6; *p <* 0.001), weight loss higher than 10% (HR = 2.38; *p =* 0.024) and miR-200c overexpression (HR = 2.24; *p =* 0.028) in the model. Residual disease, Eastern Cooperative Oncology Group (ECOG) performance status and age were not independent prognostic factors for OS.

**Table 4:** Multivariate analyses (n = 52)

	**Wald**	***p*value**	**Hazard ratio**	**95% CI**	
**Progression-free survival (PFS)**					
**Stage IV disease**	7.805	0.005	5.52	1.665	18.285
**High miR-200c**	4.835	0.028	2.27	1.093	4.712
**Residual disease (R) status**	5.195	0.023	4.29	1.226	14.993
**Overall survival (OS)**					
**Stage IV disease**	20.469	0.000	8.60	3.385	21.831
**High miR-200c**	4.827	0.028	2.24	1.091	4.614
**Weight loss > 10%**	5.074	0.024	2.38	1.119	5.048

The levels of miR-200c were considered high when the relative expression level was above the mean. Residual disease (R) was categorised as R0 when no residual disease was present and as R1-2 when microscopic or macroscopic residual disease was found.

To further explore the relationship between miR-200c expression levels and outcomes, we estimated the hazard ratios associated with the miR-200c level as a continuous variable by performing Cox multivariate regression models. Concordant results were achieved for the prediction of PFS, considering miR-200c expression levels as a continuous variable (HR = 1.004; 95% CI; *p =* 0.045) in the multivariate Cox model including stage IV and residual disease. Likewise, the risk of death was higher with increasing miR-200c relative blood expression levels (HR = 1.007; 95% CI, 1.003 to 1.012; *p =* 0.003) independent of stage and weight loss.

## Discussion

Accumulating reports have indicated that miRNAs are detectable in blood and that circulating miRNAs have the potential to be new biomarkers in patients with different diseases including cancer. Circulating miRNAs must demonstrate different hallmark characteristics to considered reliable biomarkers [15,39]: (i) stable and readily quantifiable in clinical samples; (ii) expressed by cancer cells at moderate or high levels; (iii) present at undetectable or very low levels in specimens from individuals without cancer; (iv) provide a predictive or prognostic clinical information; and (v) exhibit biological functions mechanistically linked to tumour progression.

Several studies have explored the use of miRNA expression levels in gastric tissues, sera and plasma samples to improve the diagnosis or prediction of GC [40-46]. Most reports focused on the diagnostic potential of quantifying miRNAs in blood; however, data regarding their possible prognostic role in solid tumours including GC are limited, as shown in Additional file 1, Table S3.

There are two main observations in the present study. First, miR-200c levels in the blood were significantly increased in GC patients compared with controls. These increased values were highly specific for a GC diagnosis and were associated with disease stage. Second, the blood expression levels of a single microRNA, miR-200c, provided prognostic information for patients with GC independent of a comprehensive panel of other established clinical predictors.

The miRNA-200 cluster has been shown to regulate the epithelial-mesenchymal plasticity that may be crucial at different stages of metastasis through direct targeting of the *ZEB–cadherin 1* axis [21-25]. However, *in vitro* and functional studies have yielded conflicting results regarding the net effect of miR-200 deregulation in the metastatic process [47-50]. Recent reports have indicated that tumour colonisation at metastatic sites might be enhanced by the expression of miR-200c. The xenograft model data have suggested that although miR-200 expression can hinder the intravasation of tumour cells, those that reach the circulation may be more proficient at colonising distant organs [47,48]. Our findings are consistent with these experimental data and with the clinical correlations observed between the up-regulation of miR-200c in tumours and poor prognosis in individuals with colorectal adenocarcinoma [51], oesophageal squamous cell carcinoma [52] and breast cancer [48,53].

In spite of the growing evidence highlighting its relevance in various cancers, very few studies have systematically explore the role of the miR-200 family in GC. MiR-141 was significantly down regulated in gastric cancer tissues compared with pair-matched adjacent non-tumour tissues [54,55]. Nevertheless, a recent report [56] found that miR-200a and miR-141 were significantly overexpressed in gastric cancer compared with those in normal gastric tissue. In addition, high miR-200a tumour expression was associated with a poor OS. Kurashige et al. have recently shown [57] that the downregulation of miR-200b in GC was associated with diffuse histologic type, depth of tumor, tumor size, lymph node metastasis, and lymphatic invasion. The upregulation of miR-200b was correlated with increased E-cadherin and low ZEB2. However, there were no differences in the tumour expression of miR-200c among histological types or other clinicopathological parameters.

To ascertain whether the miR-200 family expression profile can differ between GC and non-tumour mucosa and to analyse the association among miR-200a, 200b, 200c and miR-141 and histological characteristics, we used a large, public microarray database. The results of our *in silico* analyses demonstrated that the expression of miR-200a, -b, -c and miR-141 were similar in non-tumour gastric mucosae and gastric tumour tissue. Furthermore, miR-200a, -b, -c and miR-141 were not differentially expressed between intestinal and diffuse types of gastric carcinoma. In that sense, the miR-200 signature in GC was validated on an external data set. In our study, as shown in Table 2, there were no significant differences in the blood levels of miR-200c among histological types or other clinicopathological parameters. Similar data have been recently reported [57]. These findings suggest that elevated blood miR-200c levels can be detected throughout the wide spectrum of gastric adenocarcinomas and therefore underscore its potential role as a clinical biomarker.

However, tumour or cellular miRNA-expression patterns can differ from miRNA patterns released into the blood [58,59]. In addition, potential differences in the microRNAs expression profile between primary tumours and corresponding CTC or matching clinical metastases have not been systematically investigated. In that sense, the miR-200-a, -b and -c and miR-429 levels were increased in lung metastases compared to primary breast tumours [48]. Also, the expression of miR-200c/miR-141 cluster was significantly upregulated in liver metastasis from colorectal cancer, as compared with that in primary tumours [50]. Thus, circulating miRNAs may not always be directly associated with the changes occurring in primary tumor tissues.

When we considered the different reports regarding the potential diagnostic and clinical relevance of the blood-borne miRNA expression in cancer, a considerable degree of inter-study heterogeneity was noticed. Differences in the detection and quantification methods (microarrays, qRT-PCR and high-throughput sequencing technology), the types and numbers of miRNAs evaluated (pre-miRNA or mature form, expression profile or a single marker) and sample sources and timing (serum, plasma or blood cells obtained pre- or post-operatively), as well as in the clinical and pathological data of the included patients ought to be considered as potential causes of heterogeneity.

At present, there is no agreement on the most advantageous source from which to isolate circulating miRNA and the use of serum or plasma over whole blood for systemic miRNA analysis is debatable. One of the crucial problems is the efficient and reproducible extraction of small amounts of miRNA from plasma or serum. Therefore, higher yields of miRNAs have been consistently obtained from whole blood samples compared with matched serum or plasma samples and lower quantification cycles were performed in whole blood compared with matched serum and plasma samples in qRT-PCR experiments [60].

Recent reports have indicated that blood cells are major contributors of circulating miRNA [61]. Hence, one can hypothesise that increased levels of expression of epithelial-specific miRNAs in blood, including miR-200c, might indicate the circulation of tumour cells. However, the origins of circulating miRNAs are not yet clearly understood. In theory, analysis of miRNAs obtained from whole blood may be advantageous, detecting not only those miRNA derived from circulating blood cells comprising tumour cells but also those secreted in subcellular particles such as exosomes or those associated with RNA binding proteins and diverse tissues [62,63].

Any PCR-based technique still has the disadvantage of potentially detecting minimal amounts of miRNA expression in a non-disease-specific manner. Some of the proposed miRNA cancer biomarkers have been found to be highly expressed in one or more blood cell types and plasma levels of these miRNA have been correlated to blood cell counts [64]. Pritchard et al. reported that miR-200c was found in the blood and blood cells of controls, with the highest expression in neutrophils. However, patients with diverse metastatic cancer and severely ill conditions that could be considered as confounding factors were included in this study as “controls”. Conversely, we did not find any correlation between miR-200c levels and neutrophil counts in our series. In addition, miR-200c levels did not differ in subgroups defined according to neutrophil counts.

From a clinical perspective, assessment of miRNAs in the PB obtained after definitive loco-regional treatment reflects the “minimal residual disease” status that might better predict the clinical behaviour and/or therapeutic response. Postoperative sampling time combines, in theory, the baseline level of CNA, the potential release of CTC due to the surgical manipulation and the rapid death of in transit cells within the blood stream but with reduced survival ability. Our study shows that increased miR-200c levels are detected even in patients with very low tumour burdens (i.e., early-stage disease and after potentially curative R0 surgical resections).

Remarkably, we found that levels of miR-200c measured in the PB of GC patients independently correlate with OS and PFS. A clear clinical association of the expression levels of a single circulating miRNA (miR-200c) with poor survival outcomes indicated by multivariate analysis has been demonstrated. However, large prospective and follow up studies will be necessary in the near future to confirm the clinical relevance of circulating miRNAs, including miR-200c, as independent prognostic indicators for cancer.

## Conclusions

Beyond confirming initial reports, our study yielded the following evidence: (i) epithelial-derived miRNAs can be quantified in the whole-blood; (ii) the blood levels of a single epithelial and tumour-expressed miRNA, miR-200c, can distinguish, with significant specificity and sensitivity, patients with GC from healthy controls and (iii) remarkably, increased expression levels of miR-200c in blood were significantly associated with poor progression-free and overall survivals. Our study indicates unique results on its potential prognostic value that provide a firm basis for further investigation of miRNAs as blood-based cancer predictive and prognostic biomarkers.

## Abbreviations

(GC): Gastric cancer; (CTC): Circulating tumour cells; (CNA): Circulating nucleic acids; (EMT): Epithelial-to-mesenchymal transition; (PB): Peripheral venous blood; PCR (qRT-PCR): Quantitative reverse transcription; (Cq): Quantification cycle; (REST): Relative expression software tool; (ROC): Receiver operating characteristic curve; (AUC): Area under the curve; (PFS): Progression-free survival; (OS): Overall survival; (SEM): Standard error of the mean; (ECOG): Eastern cooperative oncology group.

## Competing interests

The authors declare that they have no competing interests.

## Authors' contributions

MVA conceived the study, participated in its design and drafted the manuscript. MBC, MH, VM and IS performed the molecular analyses. MR, LMAA, MQV, ACM and LC made substantial contributions to data acquisition. MVA, VM, MBC, JFT and AF made substantial contributions to data analyses and interpretation. MVA and VM performed the statistical analyses. MJLP and PID reviewed the histological samples. All authors read and approved the final manuscript.

## Supplementary Material

Additional file 1 **Figure S1.** Box plots of the miR-200 s family of microRNAs, miR-148a and miR-21 expressions in gastric cancer samples and normal gastric mucosae. Tissue miRNA concentrations were significantly lower for miR-148a (*p* < 0.0001) whereas miR-21 was significantly higher (*p* < 0.0001) in the gastric cancer samples compared to those in normal gastric mucosae. MiR-200 s were not differentially expressed in the paired non-tumour mucosa and cancer samples. MiR-148a and miR-21 were among the differentially expressed microRNAs in gastric cancer signature as defined by Ueda T, et al. The upper and lower limits of the boxes and the lines inside the boxes indicate the 75th and 25th percentiles and the median respectively. The upper and lower horizontal bars denote the 90th and 10th percentiles respectively. **Table S1**. MiR-200 s family of microRNAs, miR-148a and miR-21 expressions in the gastric cancer samples compared to those in normal gastric mucosae. **Figure S2.** Box plots of the miR-200 s family of microRNAs, miR-148a and miR-21 concentrations in gastric cancer samples according to histological type: diffuse or intestinal. Tissue miRNA concentrations were significantly higher for miR-148a (*p* = 0.004) and miR-21 (*p* = 0.011) in the diffuse type compared to intestinal type. MiR-200 s were not differentially expressed according to histological type. MiR-148a and miR-21 were among the differentially expressed microRNAs in gastric cancer signature as defined by Ueda T, et al. The upper and lower limits of the boxes and the lines inside the boxes indicate the 75th and 25th percentiles and the median respectively. The upper and lower horizontal bars denote the 90th and 10th percentiles respectively. **Table S2.** MiR-200 s family of microRNAs, miR-148a and miR-21 expressions in the gastric cancer samples according to histological type: diffuse or intestinal. **Table S3.**. Studies assessing miRNAs expression in blood among gastric cancer patients. Click here for file
