# Supplementary material for: Circulating miR-200c as a diagnostic and prognostic biomarker for gastric cancer
Source: J Transl Med. 2012 Sep 6;10:186. doi: 10.1186/1479-5876-10-186 (PMC3494541; doi:10.1186/1479-5876-10-186)
Supplement: Additional file 1 — Figure S1. Box plots of the miR-200 s family of microRNAs, miR-148a and miR-21 expressions in gastric cancer samples and normal gastric mucosae. Tissue miRNA concentrations were significantly lower for miR-148a (p < 0.0001) whereas miR-21 was significantly higher (p < 0.0001) in the gastric cancer samples compared to those in normal gastric mucosae. MiR-200 s were not differentially expressed in the paired non-tumour mucosa and cancer samples. MiR-148a and miR-21 were among the differentially expressed microRNAs in gastric cancer signature as defined by Ueda T, et al. The upper and lower limits of the boxes and the lines inside the boxes indicate the 75th and 25th percentiles and the median respectively. The upper and lower horizontal bars denote the 90th and 10th percentiles respectively. Table S1. MiR-200 s family of microRNAs, miR-148a and miR-21 expressions in the gastric cancer samples compared to those in normal gastric mucosae. Figure S2. Box plots of the miR-200 s family of microRNAs, miR-148a and miR-21 concentrations in gastric cancer samples according to histological type: diffuse or intestinal. Tissue miRNA concentrations were significantly higher for miR-148a (p = 0.004) and miR-21 (p = 0.011) in the diffuse type compared to intestinal type. MiR-200 s were not differentially expressed according to histological type. MiR-148a and miR-21 were among the differentially expressed microRNAs in gastric cancer signature as defined by Ueda T, et al. The upper and lower limits of the boxes and the lines inside the boxes indicate the 75th and 25th percentiles and the median respectively. The upper and lower horizontal bars denote the 90th and 10th percentiles respectively. Table S2. MiR-200 s family of microRNAs, miR-148a and miR-21 expressions in the gastric cancer samples according to histological type: diffuse or intestinal. Table S3.. Studies assessing miRNAs expression in blood among gastric cancer patients. [file 1479-5876-10-186-S1.doc]

**Supplementary Material**

**Figure S1.** Box plots of the miR-200s family of microRNAs, miR-148a and miR-21 expressions in gastric cancer samples and normal gastric mucosae. Tissue miRNA concentrations were significantly lower for miR-148a (*p*<0.0001) whereas miR-21 was significantly higher (*p*<0.0001) in the gastric cancer samples compared to those in normal gastric mucosae. MiR-200s were not differentially expressed in the paired non-tumour mucosa and cancer samples. MiR-148a and miR-21 were among the differentially expressed microRNAs in gastric cancer signature as defined by Ueda T, et al. The upper and lower limits of the boxes and the lines inside the boxes indicate the 75th and 25th percentiles and the median respectively. The upper and lower horizontal bars denote the 90th and 10th percentiles respectively.

**Figure S2.** Box plots of the miR-200s family of microRNAs, miR-148a and miR-21 concentrations in gastric cancer samples according to histological type: diffuse or intestinal. Tissue miRNA concentrations were significantly higher for miR-148a (*p*= 0.004) and miR-21 (*p*=0.011) in the diffuse type compared to intestinal type. MiR-200s were not differentially expressed according to histological type. MiR-148a and miR-21 were among the differentially expressed microRNAs in gastric cancer signature as defined by Ueda T, et al. The upper and lower limits of the boxes and the lines inside the boxes indicate the 75th and 25th percentiles and the median respectively. The upper and lower horizontal bars denote the 90th and 10th percentiles respectively.

**Table S1.** MiR-200s family of microRNAs, miR-148a and miR-21 expressions in the gastric cancer samples compared to those in normal gastric mucosae

| ID | logFC | AveExpr | t | p.Value | adj.p.Val | B |
| --- | --- | --- | --- | --- | --- | --- |
| hsa-mir-148aNo1 | 1.8297 | 9.1140 | 11.5895 | 0.0000 | 0.0000 | 42.0248 |
| hsa-mir-21No1 | -0.8297 | 12.3278 | -8.9058 | 0.0000 | 0.0000 | 25.1241 |
| hsa-mir-200a-prec | -0.0834 | 9.8751 | -0.5747 | 0.5663 | 0.7652 | -7.2715 |
| hsa-mir-200bNo1 | -0.0695 | 9.9517 | -0.4916 | 0.6236 | 0.7652 | -7.3158 |
| hsa-mir-141-precNo1 | -0.0501 | 9.1793 | -0.4718 | 0.6377 | 0.7652 | -7.3254 |
| hsa-mir-200cNo1 | 0.0436 | 10.3469 | 0.2830 | 0.7775 | 0.7775 | -7.3967 |

**Table S2. MiR-200s family of microRNAs, miR-148a and miR-21 expressions in the gastric cancer samples according to histological type: diffuse or intestinal**

| ID | logFC | AveExpr | t | p.Value | adj.p.Val | B |
| --- | --- | --- | --- | --- | --- | --- |
| hsa-mir-148aNo1 | -0.696 | 8.307 | -2.955 | 0.004 | 0.021 | -1.987 |
| hsa-mir-21No1 | -0.306 | 12.744 | -2.566 | 0.011 | 0.033 | -2.965 |
| hsa-mir-200cNo1 | 0.285 | 10.333 | 1.756 | 0.081 | 0.162 | -4.581 |
| hsa-mir-141-precNo1 | 0.189 | 9.229 | 1.422 | 0.157 | 0.235 | -5.076 |
| hsa-mir-200a-prec | 0.082 | 9.940 | 0.476 | 0.635 | 0.762 | -5.922 |
| hsa-mir-200bNo1 | 0.045 | 10.011 | 0.262 | 0.793 | 0.793 | -5.997 |

**List of abbreviations**

**logFC**: a positive results indicated downregulation; a negative results indicated upregulation.

**AveExpr**: Average expression in all the samples

**t**: T-test statistic

**p.value**: Unadjusted p value

**adj.p.Val**: Adjusted p value, FDR= false discovery rate, according Benjamini and Hochberg.

**B**: Expression index. A higher B value indicates a higher differential expression.

| **Table S3.** Studies assessing miRNAs expression in blood among gastric cancer patients | | | | | | | | | | | |  | |  | |  |
| --- | --- | --- | --- | --- | --- | --- | --- | --- | --- | --- | --- | --- | --- | --- | --- | --- |
|  |  | |  |  | | |  |  | |  | |  | |  | |  |
| **Author(s), year** | **Ref.** | | **Study population** | | | | | **Method qRT-PCR** | | **Source** | | **Timing** | | **Normalization** | | **miRNAs selection** |
|  |  | | **Country** | **Cases (stage I/II/III/IV)** | | | **Controls** |  | |  | |  | |  | |
| Zhou H, et al. 2010 | 40 | | China | 90 (stages NR) | | | 27 | SYBR Green | | MNC* | | Pre- and post-operative (41/49) | | U6 snRNA | | μParaflo™ microfluidic chip |
| Tsujiura M, el. 2010 | 41 | | Japan | 69 (38/13/14/4) | | | 30 | TaqMan | | Plasma | | Pre-operative | |  | | Literature and TaqMan qRT-PCR |
| Liu R, et al. 2011 | 42 | | China | 142 (29/56/48/23)** | | | 105 | TaqMan | | Serum | | Pre-operative | |  | | Solexa, Literature and TaqMan qRT-PCR |
| Liu H, et al. 2012 | 43 | | China | 40 (4/12/11/13) | | | 41 | SYBR Green | | Serum | | Pre-operative | | U6 snRNA | | Microarrays (Agilent) and qRT-PCR |
| Konishi H, et al. 2012 | 44 | | Japan | 56 (33 I-II / 23 III-IV) | | | 30 | TaqMan | | Plasma | | Pre-operative*** | |  | | 3D-Gene miRNA microarray |
| Song M-y, 2012 | 45 | | China | 68 (31 I-III/ 22 IV/ 29 NR)* | | | 68 | TaqMan | | Serum | | Pre-operative | | miR-39 | | TaqMan low-density array |
| Wang M, et al. 2012 | 46 | | China | 65 (33 I-II, 24 III, 8 IV) | | | NR | SYBR Green | | Plasma | | Pre-operative | | U6 snRNA | | Previous work (unpublished data) |
| Valladares-Ayerbes M, et al. 2012 | Present serie | | Spain | 52 (9 I-II/ 12 III /31 IV) | | | 15 | SYBR Green | | Whole blood | | Pre- and post-operative (15/37) | | U6 snRNA and 5S rRNA | | Literature, bioinformatics and qRT-PCR in cell lines |
|  |  | |  |  | | |  |  | |  | |  | |  | |  |
| NR, not reported. * Blood Mononuclear cells (MNC). **Stage distribution in the all cohort. Validation set included 142 patients. ***Pre- versus post-operative in 29 cases in validation cohort | | | | | | | | | | | | | | | |  |
|  |
| **Table S3.** Cont. | |  | |  |  |  | | |  | |  | |  | |  | |
| **Author(s), year** | | **Ref.** | |  | **Diagnostic performance** | | | | | | | | **PFS and OS Prognostic Value** | |  | |
|  | |  | | **Target(s)** | **AUC** | **95%CI** | | | **Sensitivity** | | **Specificity** | |  | |  | |
| Zhou H, et al. 2010 | | 40 | | miR-106a and miR-17 | 0.741 | 0.620-0.839 | | | 62.96 | | 80.49 | | NR | |  | |
| Tsujiura M, el. 2010 | | 41 | | miR-106b* | 0.721 | NR | | | NR | | NR | | NR | |  | |
| Liu R, et al. 2011 | | 42 | | miR-1, miR-20a, miR-27a, miR-34a, miR-423-5p | 0.879 | 0.822-0.936 | | | 80 | | 81 | | NR | |  | |
| Liu H, et al. 2012 | | 43 | | miR-378 | 0.861 | 0.766–0.928 | | | 87.5 | | 70.73 | | NR | |  | |
| Konishi H, et al. 2012 | | 44 | | miR-451 | 0.96 | NR | | | 96.0 | | 100.0 | | NR | |  | |
| Song M-y, 2012 | | 45 | | miR-221 | 0.700 | 62-78 | | | 82.4† | | 58.8† | | NR | |  | |
|  | |  | | miR-376c | 0.710 | 62-80 | | |  | |
|  | |  | | miR-744 | 0.740 | 65-82 | | |  | |
| Wang M, et al. 2012 | | 46 | | miR-17-5p | NR | NR | | | NR | | NR | | Yes, Univariate OS | |  | |
|  | |  | | miR-20a | NR | NR | | | NR | | NR | | Uni and multivariate OS | |  | |
| Valladares-Ayerbes M, et al. 2012 | | Present serie | | miR-200c | 0.715 | 0.597–0.833 | | | 65.4 | | 100 | | Yes, univariate and multivariate OS and PFS | |  | |
|  | |  | |  |  |  | | |  | |  | |  | |  | |
| PFS: Progression free survival. OS: Overall survival | | | | | |  | | |  | |  | |  | |  | |
| † Sensitivity and specificity based on the combination of miR-221, miR-744, and miR-376c | | | | | | | | | | |  | |  | |  | |
